# The complete mitochondrial genome of the carnivorous flowering plant *Nepenthes X Ventrata*

**DOI:** 10.1080/23802359.2018.1532353

**Published:** 2018-10-29

**Authors:** Eugene V. Gruzdev, Andrey V. Mardanov, Alexey V. Beletsky, Nikolai V. Ravin, Konstantin G. Skryabin

**Affiliations:** Institute of Bioengineering, Research Center of Biotechnology of the Russian Academy of Sciences, Moscow, Russia

**Keywords:** Mitochondrial genome, carnivorous plant, Nepenthes, gene transfer

## Abstract

Carnivorous plants have the ability to capture and digest small animals as a source of additional nutrients, which allows them to grow in nutrient-poor habitats. This study reports the complete mitochondrial genome sequence of a pitcher plant *Nepenthes x ventrata*. It was 520,764 bp in size with a GC content of 44.17% and contained 37 protein-coding genes, 2 pseudogenes, 18 tRNA genes and 3 rRNA genes. Four tRNA genes and the *rps11* gene were probably transferred to mitochondrion form the chloroplast genome. Phylogenetic analysis confirmed that *N. x ventrata* belongs to the order *Caryophyllales*.

Carnivorous plants are able to attract, catch and digest their preys, usually insects, and assimilate nutrients for the growth (Adamec 1997). The obtained nutrients are primary used as source of nitrogen enabling these plants to survive in oligotrophic environments. Previous studies of organelle genomes of carnivorous plants of the genus *Utricullaria* (Lentibulariaceae) revealed the loss and pseudogenization of the NAD(P)H dehydrogenase genes in the chloroplast genome (Silva et al. 2016) while the structure and gene content of the mitochondrial genome remained typical for flowering plants (Silva et al. 2017).

We determined complete sequence of the mitochondrial genome (mtDNA) of the carnivorous plant *Nepenthes x ventrata* (Caryophyllales: Nepenthaceae), a natural hybrid between *N. alata* and *N. ventricosa* (Fleming 1979). *Nepenthes* use passive pitcher-shaped traps to catch small insects and are probably the most studied carnivorous plants (Mithöfer, 2011). Pitchers of *Nepenthes* were analysed by RNA-seq and proteomics for discovery of genetic trains related to carnivory (Hatano and Hamada 2008; Wan Zakaria et al. 2016).

*N. x ventrata* plants were grown in the greenhouse of Research Center of Biotechnology RAS, Moscow, Russia (55°41'58.4″N, 37°34'54.9″E). Total genomic DNA was extracted from leaves of a single plant (accession number NEP-CB1) and sequenced using a combination of Illumina and Nanopore techniques. The sequencing of a TrueSeq DNA library on Illumina HiSeq2500 generated 2.2 Gb high-quality cleaned sequences. 1,073,754 reads with a total length of 5 Gb were obtained on MinIon (Oxford Nanopore, UK). MinIon reads were *de novo* assembled using Miniasm 0.2 (Li 2016). Raw contigs were corrected by mapping MinIon reads back to the contigs using Minimap 0.2 and calling consensus with Racon 1.3.1 (Vaser et al. 2017). The final polishing was done with Illumina reads mapping using Pilon 1.22 (Walker et al. 2014). A single contig representing mtDNA was identified based on the comparison to other plant mitogenome sequences. mtDNA annotation was performed using MITOFY (Alverson et al. 2010) with further manual correction.

The mitochondrial genome of *N. x ventrata* (GenBank accession number MH798871) is a circular DNA molecule with 520,764 bp in length and a GC content of 44.17%. The genome contains 37 protein-coding genes, including ones for NADH dehydrogenase (*nad1, 2, 3, 4, 4L, 5, 6, 7, 9*), cytochrome c oxidase (*cox1, 2, 3*), cytochrome c biogenesis (*ccmB, C, FN, FC*), succinate dehydrogenase (*sdh3, 4*), apocytochrome b (*cob*), ATP synthase (*atp1, 4, 6, 8, 9*), ribosomal proteins (*rpl2, 5* and *rps1, 3, 4, 7, 10, 11, 12, 13, 19*), maturase and membrane transporter MttB. 9 genes contained introns and 4 of them (*rps3, nad1, nad2*, *nad5*) were trans-spliced. In addition, the genome contains 18 tRNA genes coding for 15 amino acid, three rRNA genes (*rrn5,18,26*), and two pseudogenes (*rpl16* and *rps14*). Four tRNA genes and the *rps11* gene were likely acquired from the chloroplast genome; altogether plastid-like sequences accounted for ∼28 kb.

The maximum likelihood phylogenetic tree was based on concatenated sequences of exons of mitochondrial genes from *N. x ventrata* and other 42 species (Figure 1). As expected, *N. x ventrata* is phylogenetically related to other members of the order *Caryophyllales*, *Silene latifolia* and *Spinacia oleracea*.

**Figure 1.**
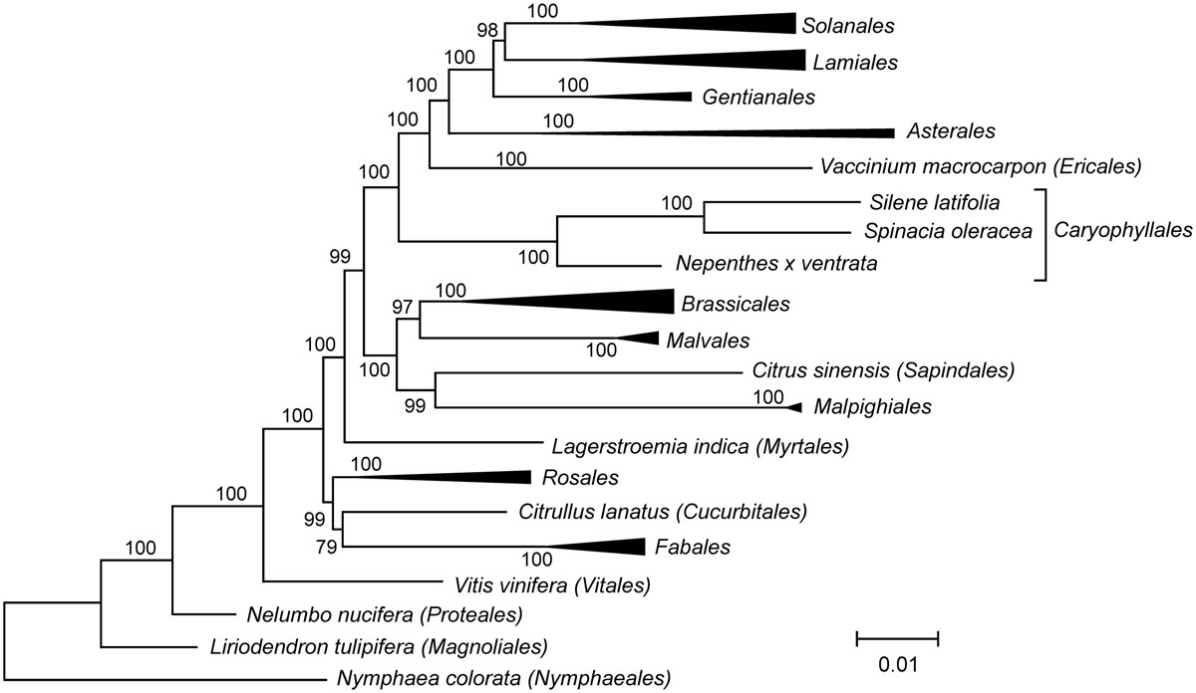
The maximum likelihood phylogenetic tree of *Nepenthes x ventrata* and 42 other angiosperm species. The tree is based on concatenated nucleotide sequences of exons of genes *atp1, atp4, atp6, atp8, atp9, ccmB, ccmC, cob, cox1, cox2, cox3, matR, nad1, nad2, nad3, nad4, nad4L, nad5, nad6, nad7*, and *nad9*, aligned using MAFFT v7.271 (Katoh et al., 2002). PhyML 3.1 (Guindon et al., 2010) was used to build the tree. Bootstrap support values are displayed on each node.
